# Stiffness regulates dendritic cell and macrophage subtype development and increased stiffness induces a tumor–associated macrophage phenotype in cancer co–cultures

**DOI:** 10.3389/fimmu.2024.1434030

**Published:** 2024-08-15

**Authors:** Carla Guenther

**Affiliations:** Department of Molecular Immunology, Immunology Frontier Research Center, Osaka University, Suita, Osaka, Japan

**Keywords:** mechanotransduction in leukocytes, dendritic cell development, integrins, macrophages, oncoimmunology, tumor microenvironment, tumor-associated macrophages, stiffness

## Abstract

Mechanical properties of tissues including their stiffness change throughout our lives, during both healthy development but also during chronic diseases like cancer. How changes to stiffness, occurring during cancer progression, impact leukocytes is unknown. To address this, myeloid phenotypes resulting from mono- and cancer co-cultures of primary murine and human myeloid cells on 2D and 3D hydrogels with varying stiffnesses were analyzed. On soft hydrogels, conventional DCs (cDCs) developed, whereas on stiff hydrogels plasmacytoid DCs (pDCs) developed. Soft substrates promoted T cell proliferation and activation, while phagocytosis was increased on stiffer substrates. Cell populations expressing macrophage markers CD14, Ly6C, and CD16 also increased on stiff hydrogels. In cancer co–cultures, CD86^+^ populations decreased on higher stiffnesses across four different cancer types. High stiffness also led to increased vascular endothelial growth factor A (VEGFA), matrix metalloproteinases (MMP) and CD206 expression; ‘M2’ markers expressed by tumor–associated macrophages (TAMs). Indeed, the majority of CD11c^+^ cells expressed CD206 across human cancer models. Targeting the PI3K/Akt pathway led to a decrease in CD206^+^ cells in murine cultures only, while human CD86^+^ cells increased. Increased stiffness in cancer could, thus, lead to the dysregulation of infiltrating myeloid cells and shift their phenotypes towards a M2–like TAM phenotype, thereby actively enabling tumor progression. Additionally, stiffness–dependent intracellular signaling appears extremely cell context–dependent, potentially contributing to the high failure rate of clinical trials.

## Introduction

Most of our knowledge on the immune system is based on chemical stimulation, such as from pathogens (pathogen associated molecular patterns, PAMPs), signaling molecules or parts of damaged cells (damage associated molecular patterns, DAMPs). However, all cell activity happens within microenvironments which present different mechanical information, such as stiffness (the extent to which materials resist deformation) and stiffness changes as a result of chronical diseases such as cancer (1–4). In addition, chronic cell damage and the release of DAMPs may contribute to cancer development (5), while DAMP levels increase in advanced tumors resulting from chemotherapy (6). An example of a PAMP is zymosan, a component of the yeast cell membrane that itself is mainly composed of β–glucan and mannan (7, 8). As such, zymosan is recognized by TLR2 (9) and C–type lectin receptors (CLRs) (10) and signaling of these receptors activates innate immune cells such as dendritic cells (11), macrophages (9) and monocytes (12).

In fact, both DAMPs and PAMPs are recognized by CLRs such as Mincle and Dectin–1 (13). However, how mechanical information affects our immune system, specifically in terms of CLR signaling, remains poorly understood.

Dendritic cells (DCs) and macrophages are central components of the immune system. These innate immune cells survey various tissues and sense abnormal ligands such as PAMPs and DAMPs via receptors such as CLRs. DCs then take up sensed antigens via adhesion receptors such as integrins during phagocytosis and migrate to the nearest lymph node to present the antigen to T cells, which initiates the adaptive immune response. Not only are integrins used for phagocytosis, but also for sensing and relaying mechanical information into the cell in order to initiate cell responses (14). One example of this is how cells typically migrate towards stiffer substrates; a process known as durotaxis (15).

Since dendritic cells and macrophages share many surface markers their distinct expression profiles are debated, while dendritic cells are regarded as more migratory than macrophages (16). However, distinct subsets for both cell types have been characterized. The broadest distinct DC subsets are conventional dendritic cells and plasmacytoid dendritic cells (16). Conventional dendritic cells are associated with migration and T cell priming and can be further distinguished into cDC1 and cDC2. pDCs are primarily associated with type I interferon production after recognizing viral or endogenous nucleic acids via TLR7 or 9 (17–20). Rodrigues et al. revealed a lineage dependent separation of cDC and pDC development, which nevertheless overlaps in common bone marrow resident progenitors. During development these progenitors diverge into Siglec H^+^/CX3CR1^-^ pre–pDCs, Siglec H^+^/CX3CR1^+^ pDC–like cDC2 progenitors and Siglec H^-^/CX3CR1^+^ pre–cDC2 (21). Both cDC and pDCs develop in the bone marrow before being released into the peripheral bloodstream and migrating to target tissues (22).

While tissue specific macrophage populations, like Alveolar macrophages and Microglia, exist, Macrophages can be broadly distinguished by their activation status. As such the proinflammatory M1 and the tissue repair associated M2 activation status are recognized (23), with M2 being heavily associated with tumor–associated macrophages (24).

The aim of this study was to elucidate how differences in tissue stiffness impact DC development. Here, it was shown that stiffness regulates DC and macrophage phenotypes beyond maturity, polarizing the mature phenotype into cDCs or pDCs. While cDCs developed more on softer substrates, high stiffnesses induced pDC development and increased CD14^+^ cell populations along with the expression of other macrophage markers. Moreover, 3D monocultures in hydrogels with different stiffnesses showed a similar tendency to develop these phenotypes. These phenotypes developed due to stiffness signals alone and also occurred and became more pronounced following cell stimulation with PAMP and DAMP CLR ligands. Differences in stiffness also affected functionality as soft substrates promoted T cell proliferation and activation, while phagocytosis was increased on stiffer substrates. These phenotypes were mediated by transcription factors such as Ikaros as well as STAT5 and STAT3 signaling. Stiffness–dependent cDC and pDC polarization also emerged in murine 3D co–cultures with melanoma cells, while both populations decreased in colon and breast cancer co–cultures. Instead, high stiffnesses induced CD14^+^ populations across all three cancer types. In human cancer co–cultures, most CD11c^+^ cells expressed CD206 on higher stiffnesses and high stiffness induced expression and production of VEGFA and MMPs in mice and humans. This suggests that stiffness contributes to switching myeloid cells towards a TAM phenotype.

Treating cell cultures with Akt Inhibitor IV reduced CD206 expression in murine CD11c^+^ cells, but not in human cells. Instead, inhibiting Akt signaling in human CD11c^+^ cells led to an increase of CD86 expression, but not in murine cells. This indicates that Akt is a central regulator of the myeloid cell phenotype, but its specific function is cell context dependent. Extracellularly the TAM phenotype coincided with increased TGF–β production in cancer co–cultures. Nevertheless, these data suggest targeting Akt in tumor–associated myeloid cells might be a valid strategy to switch an anti–inflammatory to a pro–inflammatory tumor microenvironment to induce antitumor responses and improve patient survival.

## Materials and methods

### Mice

C57BL/6 mice were purchased from CLEA Japan, Inc (Tokyo, Japan). All mice were maintained in a filtered–air laminar–flow enclosure and given standard laboratory food and water *ad libitum*. All animal protocols were approved by the Animal Care and Use Committee of the Research Institute for Microbial Diseases, Osaka University, Japan (Biken-AP-R03-17-1).

### Cell culture

Murine DCs were differentiated from isolated bone marrow via culture in RPMI + 10% FCS 1% Pen/Strep and 1% Amino Acids containing 20 ng/ml GMCSF (Biolegend, #576306) on silicone hydrogels with different stiffnesses: 0.2, 0.5, 2, 8, 16, 32, and 64 kPa (Advanced BioMatrix, # 5190-7EA). Hydrogels were coated with 100 μg/ml collagen I (Nippi, #ASC-1-100-100) or 1:100 diluted Matrigel (Falcon, #354234) according to Advanced BioMatrix’s instructions.

For 3D alginate cell cultures cells were embedded in alginate gels modified from the protocol by Elysse C. Filipe et al. (25). Briefly, four volumes of a 2% alginate (NovaMatrix, #P-1408-24 or Funakoshi, #KIM-19130-100) solution in 0.9% saline solution were mixed with two volumes of 3 mg/ml collagen I stock solution (Nippi, #ASC-1-100-100). One volume of 0.9% saline, NaOH (3.9 mM final concentration, Sigma Aldrich, #S8045), and CaCO_3_ (5 mM final concentration, Sigma Aldrich, #C4830) was added, along with bone marrow cell suspension and cancer cell suspension, where applicable. Polymerization was initiated with D-(+)-Gluconic acid δ-lactone (4.2 mg/ml final concentration, Sigma Aldrich #G4750). 1ml of gel per well was cast in 24 well plates. Gels were left to polymerize overnight at 37°C and then stiffened with a calcium chloride (Sigma Aldrich, #C1016) solution of 25 mM for 10 kPa or 50 mM for 20 kPa for 30 min at 37°C. For soft stiffness conditions, no calcium chloride was added to the polymerized gels. At the end of the culture, gels were digested with 100 μg/ml alginate lyase (Merck, #A1603-100mg) and 1 mg/ml collagenase (Sigma, #C5138) for 2 h at 37°C.

For 3D dextran cell cultures cells were embedded in dextran-based hydrogels supplied form CELLENDES according to instructions (#FG90-1 and #FG91-1). Briefly Water and 10*CB were mixed with dextran in a ratio to generate 2mM and 9mM hydrogels, after which RGD (# 09-P-001) was added to enable cell adhesion and incubated for 20min. Next, cell suspensions were mixed into the hydrogel after which either CD–Link or PEG–Link were added to initiate polymerization. CD–Link was used to generate cell cleavable hydrogels while PEG–Link generates hydrogels which cells cannot cleave. After polymerization hydrogels were washed and media was added. Cells were cultured as usual and harvested after 8 days by digesting the hydrogels with dextranase (# D10-1).

For co–cultures with cancer cell lines, the following murine cancer cell lines were used: B16F10 (melanoma, from Riken Bioresource Center), MC-38 (colon cancer), and 4T1 (breast cancer). Murine colon and breast cancer cell lines were a kind gift from the Sakaguchi lab. For human cancer cell co–cultures, the following human cell lines were used: A549 (lung cancer, ATCC, #CCL-185), MDA-MB-231 (breast cancer, ATCC, #HTB-26), and PANC-1 (pancreatic cancer, ATCC, #CRL-1469). Cancer cells were added at the beginning of the culture at a 1:10 ratio with bone marrow cells or human CD14^+^ monocytes.

### Human monocytes

Human CD14^+^ monocytes were isolated from the peripheral blood of healthy donors using lymphocyte separation solution based gradient centrifugation (Nacalai Tesque, #20828-44) followed by magnetic bead sorting utilizing anti–human CD14^+^ antibodies (Miltenyi Biotech, #130-050-201). CD14^+^ monocytes were cultured in RPMI–1640 media supplemented with 10% FCS, 0.1 mM non–essential amino acids (Gibco, #11140-050), 1 mM sodium pyruvate (Nacalai Tesque, #06977-34), 1% Pen/Strep, 0.1% Amphotericin B (Thermo Fisher Scientific, #15290026), 10 ng/ml hGM–CSF (Peprotech, #576306), and 10 ng/ml hIL–4 (Peprotech, #574004) for eight days. Donors had given informed consent in accordance with the Declaration of Helsinki prior to blood donation. Donors consisted of five females and four males aged 25–39. The institutional review board of Osaka University approved the blood draw protocols for healthy individuals (approval number 29-4-10).

### Stimulation and inhibitor

Dendritic cells were stimulated or inhibited on day 9 of the culture for 24 h. Cell stimulation was done with 100 μg/ml zymosan (Sigma, #Z4250-1G), 100 μg/ml depleted zymosan (Nacalai, #59008-84), 20 ng/ml LPS (Sigma, #L4516), 10 μg/ml trehalose 6,6’–dimycolate (Sigma, #T3034), 10 μg/ml β–glucosylceramide (Avanti Polar Lipids, #860549P-5mg) or 500ng/ml IL–12/IL–23 p40 (biotechne, #499-ML-025/CF). To inhibit Akt, cell cultures were treated with 1.2 μM Akt Inhibitor IV (Cayman, #15569) for 24 h.

### RNA sequencing

For RNA sequencing, cells were directly lysed on the hydrogels with ice–cold Trizol (Thermo, #15596026). Stimulated cells were treated 20 h before lysis. Lysates were collected and submitted to the NGS service of the Osaka University. Briefly, total RNA was extracted using a RNeasy Mini kit (QIAGEN) and library preparation was performed using a TruSeq–stranded mRNA sample prep kit (Illumina, San Diego, CA, USA) according to the manufacturer’s instructions. Whole–transcriptome sequencing was then performed with the RNA samples using the Illumina HiSeq 2500, HiSeq 3000 or NovaSeq 6000 platforms in the 75– or 101–base single–end mode. Sequenced reads were mapped to the mouse reference genome sequences (mm10) using the TopHat software, version 2.1.1 (John Hopkin’s University, Baltimore, MA, USA). The number of fragments per kilobase of exon per million mapped fragments (FPKMs) was calculated using the Cufflinks (GitHub) software, version 2.2.1. Unsupervised k–means clustering analysis was performed via the integrated Differential Expression and Pathway analysis website (http://bioinformatics.sdstate.edu/idep/). RNA–seq data was deposited at the Sequence Read Archive under the Accession number: PRJNA1082503.

### Flow cytometry

After detaching the cells, FC stain was performed (1:50, BD Pharmingen #553142). The following antibodies were used at a concentration of 1:100: CD14 (BioLegend, #123312, clone Sa14-2), CD86 (BioLegend, #105012, clone GL-1), Langerin (BioLegend, #BL144203, clone 4C7), CD317 (BioLegend, #127108, clone 129C1), CD16 (BioLegend, #158008, clone S17014E), CD205 (BioLegend, #138214, clone NLDC-145), CD11b (BioLegend, #101207, clone M1/70), CD11c (BP Pharmingen, #5538C1, clone HL3 and BioLegend, #117310, clone N418), TLR7 (BioLegend, #160004, clone A94B10), Siglec-H (BioLegend, #129603, clone 551), CD319 (BioLegend #152003, clone 4G2), STAT5p (BD Pharmingen, #612567, clone 47/Stat5), LY6C (BD Pharmingen, #553104, clone AL-21), STAT4p (Invitrogen, #17-9044-41, clone 4LURPIE), CD80 (BD Pharmingen, #553768, clone 16-10A1), Ikaros (BioLegend, #653303, clone 2A9/Ikaros), STAT1p (BioLegend, #686403, clone A15158B), STAT3p (BioLegend, #651020, clone 13A3-1), B220 (eBioscience, #11-0452-85, clone RA3-6B2) CD206 (BioLegend, #141727, clone C068C2), I-A/I-E (BioLegend, #107621, clone M5/114.15.2), CD150 (Biolegend, #115903, clone TC15-12F12.2), CD163 (Biolegend, #155305, clone S15049), STAT4 (Proteintech, #CL488-67568, clone 4A8C9), CD69 (Biolegend, #104514, clone H1.2F3), hCD16 (BD Pharmingen, #560918, clone 3G8), hCD14 (BD Pharmingen, #561029, clone M5E2), hCD317 (BD Biosciences, #566381, clone Y129), hCD11c (BD Pharmingen, #560999, clone B-ly6), hCD86 (BioLegend, #305412, clone IT2.2), and hCD206 (BioLegend, #321136, clone 15-2). Dead cells were stained with 2 μg/ml propidium iodide (Sigma, #P4170) or 7AAD (1:100, BioLegend, #420404).

Intracellular staining was done after permeabilization/fixation with the eBioscience Foxp3 transcription factor staining buffer set (Invitrogen, #00-5523-00) according to the manufacturer’s instructions. Samples were measured with an Attune NxT (Thermo Fisher Scientific, #A24858) and analyzed via FlowJo (TreeStar). T cell proliferation was analyzed via the Proliferation Plugin.

### ELISA

The following ELISA kits were used according to the manufacturer’s instructions: TNF (BD Biosciences, #558534), IL–6 (BD Biosciences, #555240), IL–2 (BD Biosciences, #555148), IL–4 (BD Biosciences, #555148), IFN–γ (BD Biosciences, #551866), VEGF_165_ (Peprotech, #900-K99), human MMP13 (R&D, #DY511), human TGF–β1 (BD Biosciences, #559119).

### T cell activation and proliferation assay

Bone marrow was isolated from three mice and cultured in GMCSF media for seven days. On day seven DCs were detached and pooled. On this day T cells were isolated from spleens and lymph nodes of three OT I and three OT II mice via magnetic beads (Miltenyi, #130-117-044, #130-117-043). T cells were stained with CellTrace™ Violet (Invitrogen, #C34557). T cells of one animal were mixed 1:1 with pooled GMCSF cultured in one well of a 6 well plate (Advanced Biomatrix hydrogel plates and plastic plates). 0.5mg/ml OVA was added to each well except one well on a plastic 6 well plate, that served as a negative control. Cells were cultured for three more days, after which CTV fluorescence was measured along with activation marker CD69. The culture supernatant was saved and used for IL–2, IL–4 and Interferon–gamma ELISAs (BD Biosciences, #555148, #555232, #551866).

### Phagocytosis assay

Dendritic cells were cultured on hydrogels as described above and at the end of the culture 100μg of pHrodo™ Green Zymosan BioParticles™ Conjugate for Phagocytosis, which become fluorescent when phagocytosed due to acidic pH environments of endosomes and lysosomes (Invitrogen, #P35365), were added to cells and incubated for 30min. Next, cells were detached and washed 5 times with 2% FCS–RPMI, after which Alexa488 fluorescence was measured. Non treated cells were used as a negative control.

### Statistics

All experiments were performed with three biological repeats and were repeated at least twice (technical repeats, one technical repeat consisted of three biological repeats), except for the RNA sequencing and unless there was no statistically significant difference observed (such as to compare stimulations) to reduce the number of animals used. To calculate the statistical significance and generate the graphs, Graph Pad Prism 9 was used. Unpaired, two–tailed Student’s t–tests or Mann–Whitney tests were used unless otherwise indicated. All p values are shown as *p < 0.05, **p < 0.01, ***p < 0.005, and ****p < 0.0001. For treatments and comparisons shown as fold change Benjamini–Hochberg procedure corrected p values were used (q), for these the values are depicted as follows: *p < 0.05 and **p < 0.0001.

## Results

### Substrate stiffness polarizes DC subtype phenotypes into cDC vs pDC in 2D and 3D

**Figure 1:**
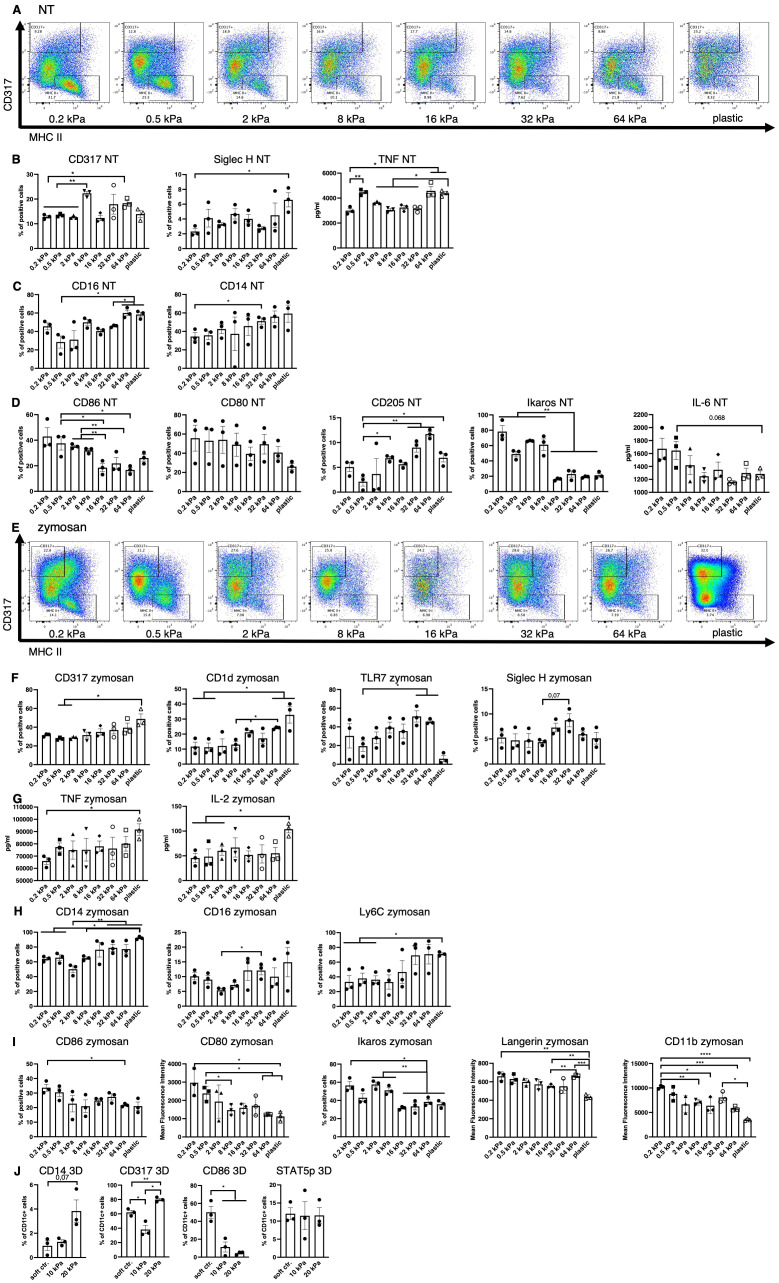
Low stiffness induces the cDC and high stiffness the pDC phenotype. **(A)** An example of flow plots depicting the shifting CD317^+^ and MHCII^+^ cell populations across the 8 stiffnesses that arose in BM derived cell cultures without stimulation (NT, not treated). **(B)** Cell populations positive for pDC associated surface markers were measured in resting cells (NT, not treated) via flow cytometry. Percentages of all living cells are shown. The production of TNF was measured via ELISA. **(C)** Cell populations positive for macrophage markers were measured in resting cells (NT, not treated) via flow cytometry. Percentages of all living cells are shown. **(D)** Soft substrates induced an increase of cell populations positive for cDC associated markers as well as IL–6 production in resting cells. Cell populations positive for cDC–associated surface markers were measured in resting cells (NT, not treated) via flow cytometry. Percentages of all living cells are shown. The production of IL–6 was measured via ELISA. **(E)** An example of flow plots depicting the shifting CD317^+^ and MHCII^+^ cell populations across the 8 stiffnesses that arose in BM–derived cell cultures after zymosan stimulation (24 h). **(F)** Cell populations positive for pDC associated surface markers were measured in zymosan–stimulated (24 h) cells via flow cytometry. Percentages of all living cells are shown. **(G)** The production of TNF and IL–2 was measured via ELISA. **(H)** Cell populations positive for macrophage surface markers were measured in zymosan–stimulated (24 h) cells via flow cytometry. Percentages of all living cells are shown. **(I)** Cell populations positive for cDC associated surface markers were measured in zymosan–stimulated (24 h) cells via flow cytometry. Percentages of all living cells are shown, for Langerin and CD11b expression levels (MFIs) are shown. **(J)** The cell populations of BM–derived cells cultured in 3D alginate/collagen hydrogels (25). Surface marker expression was measured via flow cytometry. Cell populations were first gated for CD11c in order to exclude gel particles and the graphs depict the percentage of CD11c^+^ cells. 10 kPa and 20 kPa were stiffened by addition of CaCl_2_ and soft controls were not stiffened (soft ctrl.). (**A–J)** All experiments were performed with three biological repeats per technical repeat and at least two technical repeats in total. One representative technical repeat is shown in each graph and statistics are based on three biological repeats per condition depicted in the graph. All p values are shown: *p < 0.05; **p < 0.01; ***p < 0.005; ****p < 0.0001. All error bars are shown as SEM.

To investigate the stiffness impact on DC phenotypes, bone marrow (BM) cells were differentiated into DCs using granulocyte macrophage colony stimulating factor (GMCSF). Cells were cultured on commercially available silicone hydrogels with different stiffnesses (0.2 kPa, 0.5 kPa, 2 kPa, 8 kPa, 16 kPa, 32 kPa and 64 kPa which roughly correlate with different tissues (e.g. brain, lung, kidney, muscle, eye and bone). As a positive control conventional tissue culture plastic was used. To present the same chemical surface to the cells, hydrogels and plastic were coated with 100 μg/ml collagen. This culture system resulted in at least 60% CD11c^+^ cell populations across all stiffnesses (**Supplementary Figure 1A**). Importantly, different DC phenotypes were observed across the different stiffnesses even without further stimulation, with clear populations emerging as shown in **Figure 1A** when analyzed via flow cytometry (gating strategy shown in **Supplementary Figure 1B**).

On stiff substrates percentages of CD317^+^ and Siglec–H^+^ increased significantly (**Figure 1B**), while B220 expression level showed a tendency to be increased (**Supplementary Figure 1C**) and TNF production increased, all of which is associated with the pDC phenotype (**Figure 1B**). There was a significant increase in TNF production, from 0.2 to 0.5 kPa, indicating that even extremely small stiffness changes can significantly impact the DC phenotype. Additionally, percentages of CD14^+^ and CD16^+^ cells were increased (**Figure 1C**) on stiffer substrates, as well as CD319, which is expressed by NK cells and a minor monocyte subset (**Supplementary Figure 1D**) (26). On soft substrates, CD80^+^, CD86^+^ and Ikaros^+^ cell populations and IL–6 production were increased (**Figure 1D**), which is associated with cDCs and decreased integrin signaling (27, 28). However, populations positive for cDC marker CD205 were decreased on softer substrates (**Figure 1D**).

Following stimulation with zymosan, these phenotypes did not disappear (**Figure 1E**). A seemingly linear increase in CD317^+^ cells as well as an increase of CD1d^+^, TLR7^+^ and Siglec–H^+^ (trend, not significant) populations (**Figure 1F**) was observed on higher stiffnesses, along with an increased production of IL–2 and TNF compared to the extreme stiffness of plastic (**Figure 1G**). It is noteworthy, that IL–2 secretion was increased at 8 kPa compared to 0.2 and 0.5 kPa and TNF secretion was increased on 16 and 64 kPa compared to 0.2 kPa. Stiffer substrates also gave rise to increased populations of cells positive for the macrophage markers CD14, CD16, and Ly6C (**Figure 1H**) and NK marker CD319^+^ (**Supplementary Figure 1E**). On soft substrates CD86^+^ and Ikaros^+^ cell populations increased along with CD80, Langerin and CD11b expression levels (**Figure 1I**). Furthermore, these phenotypes arose regardless of the ligand used for stimulation (DAMP or PAMP), as shown for the example of CD86 and CD80 (**Supplementary Figure 1F**).

To validate these results in a more *in vivo–*like model, bone marrow derived DC (BMDC) monocultures in 3D alginate/collagen hydrogels were done, resulting in similar decreases in CD11c^+^/CD86^+^ cells on high stiffnesses, while CD11c^+^/CD317^+^ and CD11c^+^/CD14^+^ cell populations increased on high stiffnesses (**Figure 1J**). There was no difference between CD11c^+^/STAT5p^+^ populations across stiffnesses. Alginate is a polysaccharide and as such was shown to induce LPS–like immune responses in macrophages (29) and could thus artificially skew DC development. To address this alginate effect, an alternative dextran–based 3D culture system was used to confirm the validity of the alginate results. While Dextran is taken up by innate immune cells via CD206, it is considered an inert ligand [reviewed in (30)]. In this dextran system CD11c^+^/CD80^+^ cell populations were increased on the softer 2M gels while CD11c^+^/TLR7^+^ and CD11c^+^/CD14^+^ cell populations increased on the stiffer 9M gels (**Supplementary Figure 1G**). These results indicate these cell populations arise due to differences in stiffness independent of chemical stimuli from the microenvironment.

**Figure 2:**
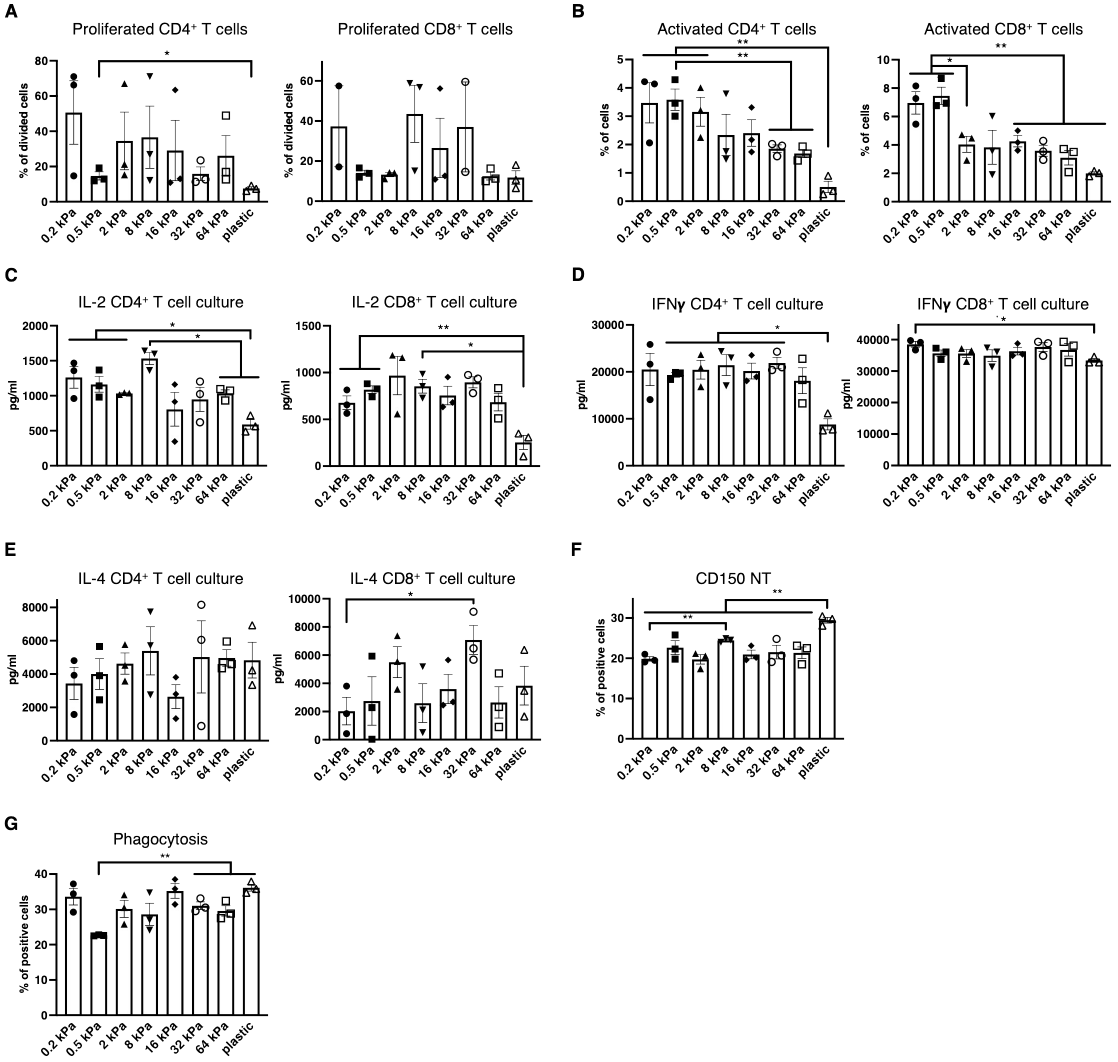
Low stiffness promotes T cell proliferation and Th1 polarization while high stiffness promotes phagocytosis. **(A)** Percentages of proliferating CD4^+^ and CD8^+^ T cells in BMDC:T cell cocultures stimulated with Ovalbumin as calculated via FlowJo’s Proliferation plugin based on CellTrace™ Violet fluorescence. **(B)** Percentages of activated T cells were measured as CD69^+^ cells. **(C)** IL–2 **(D)** Interferon–gamma and **(E)** IL–4 secretion were measured from BMDC:T cell coculture supernatants. **(F)** Bar graphs depicting CD150 expression of resting BMDC cultures measured via flow cytometry. **(G)** Bar graphs depicting phagocytosis measured as BMDCs that had taken up zymosan linked BioParticles. (**A–G)** All experiments were performed with three biological repeats per technical repeat and two technical repeats in total. One representative technical repeat is shown in each graph and statistics are based on three biological repeats per condition depicted in the graph. All p values are shown: *p < 0.05; **p < 0.01. All error bars are shown as SEM.

Next, it was investigated if the changes in surface marker expression coincided with functional consequences across the stiffnesses. To test this, T cells were isolated from OT II and OT I mice, labeled, co–cultured with BMDCs and stimulated with Ovalbumin. Percentages of proliferated CD4^+^ T cells were increased on softer substrates, while no differences were observed for CD8^+^ T cells (**Figure 2A**). Both CD4^+^ and CD8^+^ T cells were activated at higher percentages on softer substrates and also produced more IL–2 on softer substrates (**Figures 2B, C**). It is noteworthy, that IL–2 levels seemed increased on intermediate stiffnesses in the CD8^+^ T cell cocultures. Interferon–gamma (IFN–γ), associated with Th1 was secreted at the highest level out of the analyzed cytokines and secretion level were relatively similar across the stiffnesses but declined on stiffer substrates, especially plastic (**Figure 2D**). IL–4 secretion seemed to increase with stiffness, but seemingly not in a linear fashion, as IL–4 level at 16 kPa were lower than at 2 or 32 kPa (**Figure 2E**). Conversely to this functional data, CD150^+^ populations were increased on individual stiffnesses like 8 kPa and plastic (**Figure 2F**) and thus did not mirror T cell activation despite this marker being associated with T cell activation. Next, phagocytosis was analyzed by adding zymosan linked Bioparticles to cell cultures and measuring resulting fluorescence after 30 minutes of incubation. While phagocytosis was relatively high on 0.2 kPa, more cells had taken up bioparticles on stiffer substrates compared to 0.5 kPa (**Figure 2G**). Thus, on a functional basis, stiffness promotes different functions across the stiffness gradients, with softer substrates promoting T cell proliferation, activation and Th1 polarization, while stiffer substrates might somewhat promote Th2 polarization and also phagocytosis.

**Figure 3:**
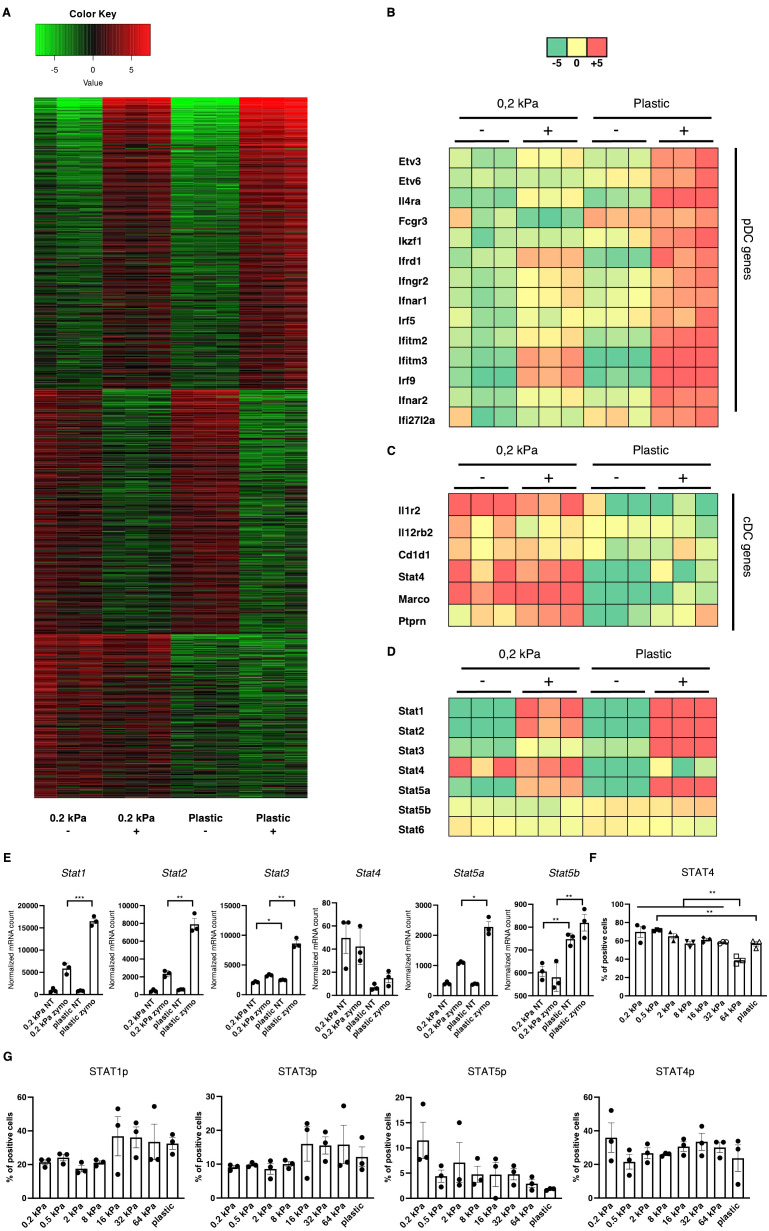
Stiffness–induced cDC and pDC phenotypes are mediated by STAT signaling. **(A)** Heatmap depicting global gene expression changes between DCs cultured on 0.2 kPa hydrogels (+/- zymosan) and DCs cultured on plastic (+/- zymosan). Zymosan stimulation was done for 20 h. **(B)** Genes associated with the pDC phenotype were upregulated on plastic, **(C)** while genes associated with the cDC phenotype were upregulated on 0.2 kPa hydrogels. **(D)** Heatmap depicting STAT gene expression changes. **(E)** Bar graphs depicting normalized mRNA counts of STAT genes. **(F)** Bar graphs depicting percentages of STAT4^+^ populations at the end of BMDC cultures and after overnight stimulation with IL–12/IL23 measured via flow cytometry. **(G)** Bar graphs depicting STAT phosphorylation at the end of BMDC cultures measured via flow cytometry. The percentages for all living cells are shown. RNAseq experiments were performed using three biological repeats per condition. Flow cytometry experiments were performed with three biological repeats per technical repeat and at least two technical repeats in total. One representative technical repeat is shown in each graph. Three biological repeats per condition were used for the statistical analysis. All p values are shown: *p < 0.05; **p < 0.01; ***p < 0.005. All error bars are shown as SEM.

To identify the central regulators governing these phenotypical changes, RNA sequencing was performed on 2D cultures derived from the two most extreme stiffnesses, 0.2 kPa and plastic both with and without zymosan stimulation. Beyond the primary zymosan effect, there was also a smaller set of genes persistently upregulated on soft substrates regardless of zymosan stimulation (**Figure 3A**). Upon further analysis, multiple interferon–associated genes were discovered to be upregulated on zymosan–induced plastic as well as several transcription factors, such as *Etv3*, *Etv6*, and *Il4ra* (**Figure 3B**). On 0.2 kPa gels, multiple genes associated with cDCs were upregulated, such as *il12r*, *il12rb2, Cd1d1, Marco*, and *Ptprn* (**Figure 3C**). As DC phenotypes are regulated by signal transducer and activator of transcription (STAT) signaling (31), expression of these transcription factors was also analyzed (**Figure 3D**). While zymosan treatment led to *Stat1* and *Stat2* upregulation compared to unstimulated controls, there was also a significant increase on plastic compared to 0.2 kPa gels for both genes (**Figures 3D, E**). This pattern was also observed for *Stat3* and *Stat5a*, visible in the heatmap (**Figures 3D, E**). Notably, *Stat4* was upregulated on 0.2 kPa and downregulated on plastic. To further investigate STAT4 expression, BMDC cultures were stimulated with IL–12/IL–23, a cytokine known to stimulate the STAT4 pathway. This stimulation led to increased STAT4^+^ populations on softer substrates, which declined in a linear fashion with increased stiffness (**Figure 3F**).

STAT signaling is predominantly mediated by phosphorylation (32). Thus, to confirm the RNA results, we investigated STAT phosphorylation via flow cytometry (**Figure 3G**). While no significant differences were observed, several trends emerged. First, STAT1p and STAT3p were predominantly elevated on stiffer substrates, suggesting their involvement in mediating the pDC phenotype, mirroring previous reports (31, 33). However, Stat5 phosphorylation decreased on stiffer substrates. While the cDC phenotype has been shown to be mediated by Ikaros (**Figures 1C**, **F**) (28), others studies indicated that STAT5 suppresses the pDC phenotype (34). Lastly, STAT4 phosphorylation did not mirror the RNA expression data, as no consistent stiffness dependent effect on STAT4p could be observed. It is believed there is currently no established role for STAT4 in DC development, however Remoli et al. reported STAT4’s interferon 1–dependent presence in mature DCs (35), which is in line with the results presented here. It is important to note that here, STAT phosphorylation was analyzed at the end of the culture and these results represent a baseline in these cells.

### High stiffness induces a macrophage phenotype in 3D cancer co–cultures

**Figure 4:**
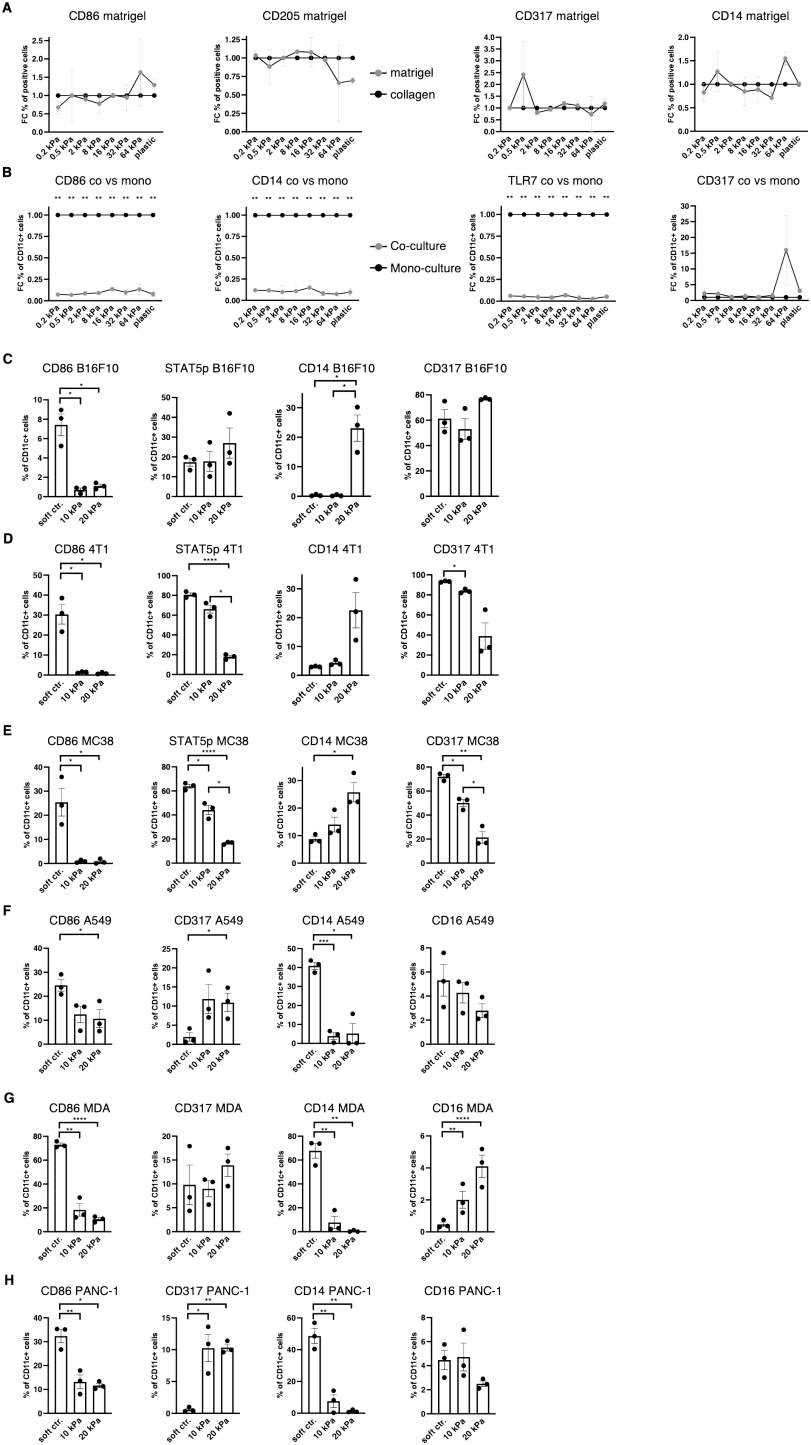
Increased stiffness in cancer co–cultures contributes to cDC and pDC phenotype dysregulation. **(A, B)** For all graphs, individual control (collagen or monoculture) percentages were set as 1 for each stiffness, upon which the corresponding fold change was calculated. **(A)** Surface marker expression of resting BMDC cultures on collagen or matrigel measured via flow cytometry. The percentages of all living cells are shown. **(B)** Surface marker expression of resting BMDC cultures compared to cancer co–cultures (B16F10). Cell percentages are based on CD11c^+^ cell populations and monocultures were set as a baseline. **(C)** Cell percentages of CD11c^+^ cell populations from B16F10 co–cultures. **(D)** 4T1 (breast cancer) **(E)** and MC-38 (colon cancer) co–cultures are shown. **(F)** The surface marker expression of human CD14^+^ cell co–cultures with A549 (lung cancer), **(G)** MDA-MB-231 (short MDA, breast cancer), **(H)** and PANC-1 (pancreatic cancer). (**F–H)** Cell percentages of CD11c^+^ cell populations are shown. (**C–H)** 10 kPa and 20 kPa were stiffened by addition of CaCl_2_ and soft controls were not stiffened alginate/collagen hydrogels. All experiments were performed using three biological repeats per technical repeat and at least two technical repeats in total. One representative technical repeat is shown in each graph. Three biological repeats per condition were used for the statistical analysis. **(A, B)** All statistical differences are depicted as the Benjamini–Hochberg procedure corrected p values: *p < 0.05; **p < 0.0001. (**C–H)** All p values are shown: *p < 0.05; **p < 0.01; ***p < 0.005; ****p < 0.0001. All error bars are shown as SEM.

During cancer progression, immune cells including DCs frequently become dysregulated and the stiffness of the tissue changes, especially due to stromal changes in extracellular matrix (ECM) production (36). Melanoma specifically has been shown to increase skin stiffness, from 2–3 kPa to around 25 kPa (4). cDCs as well as pDCs have been found to be beneficial for anti–tumor responses to breast cancer (37), while CD14^+^ cells are associated with immunosuppression across cancer types (38). It was thus hypothesized that a tumor–associated stiffness increase contributes to the dysregulation of innate immune cells such as DCs, macrophages, and their progenitors. In tumor progression, many molecules are produced, some of which are directly responsible for the stiffness of tumors, such as the tumor ECM (36). To test if the tumor ECM overrides the stiffness effect, Matrigel, a commercially available tumor–derived basement membrane, was used to coat hydrogels and these were compared with collagen–coated hydrogels. Steady–state cultures resulted in no significant changes in populations, as shown in the CD86^+^, CD205^+^, CD317^+^ and CD14^+^ cell populations (**Figure 4A**). This indicates that tumor–derived ECM molecules do not alter the stiffness–mediated DC and macrophage phenotypes.

Next, it was investigated if cancer cells would induce different phenotypes in co–cultures. For this, B16F10 melanoma cells were added at the start of the culture and CD11c was used to distinguish DCs from melanoma cells. Surprisingly, CD11c^+^/CD86^+^, CD11c^+^/CD14^+^ and CD11c^+^/TLR7^+^ cell populations were almost completely abolished in 2D co–cultures (B16F10), while there was no change to CD11c^+^/CD317^+^ cell populations (**Figure 4B**).

Next, development of co–cultures in 3D was investigated. In 3D melanoma co–cultures CD11c^+^/CD14^+^ cells increased in 20 kPa gels while CD11c^+^/CD317^+^ cell populations remained unchanged along with CD11c^+^/STAT5p^+^ populations. By contrast, CD11c^+^/CD86^+^ cells decreased on stiffer substrates (**Figure 4C**). To determine if this was a general mechanism, it was necessary to confirm these results with two other cancer cell types. For this purpose, 3D co–cultures with breast cancer cells (4T1, **Figure 4D**) and colon cancer cells (MC38, **Figure 4E**) were established. In both cultures CD11c^+^/CD317^+^ and CD11c^+^/STAT5p^+^ decreased with stiffness, while CD11c^+^/CD86^+^ populations were only present on soft controls. Finally, CD11c^+^/CD14^+^ populations increased with stiffness. This suggests that some cancer cells can prevent CD11c^+^/CD317^+^ from developing at high stiffnesses, which would be beneficial in evading the anti–tumor pDC phenotype altogether. Ultimately, high stiffness suppressed cDC development and favored progenitors developing into CD14^+^ populations.

Finally, 3D co–culture experiments using human cells were performed to validate the murine data. Instead of using bone marrow–derived cells, CD14^+^ cells were isolated from the blood of nine healthy donors. Cultures with lung cancer (A549), pancreatic cancer (PANC–1), and breast cancer (MDA-MB-231) were established and revealed similar results to the murine cultures regarding CD11c^+^/CD86^+^ cell populations (**Figures 4F–H**). Notably, CD11c^+^/CD317^+^ were not lost on higher stiffnesses but did not exceed 15%. These results mirror the murine results, whereby most cells did not have a cDC or pDC phenotype in higher stiffnesses in cancer co–cultures. Surprisingly, CD11c^+^/CD14^+^ cell populations drastically decreased in higher stiffnesses. It was hypothesized that the initial myeloid progenitors were further developed compared to the murine BM cultures and, thus, might have differentiated into another phenotype, such as a mature macrophage phenotype. To test this hypothesis CD11c^+^/CD16^+^ cell populations were investigated, as CD16^+^ cell populations increased on stiff substrates in 2D. However, CD11c^+^/CD16^+^ cell populations did not increase at all or not to a cell population larger than 5% in higher stiffness gels.

### Tumor–associated macrophage marker expression increases with stiffness

**Figure 5:**
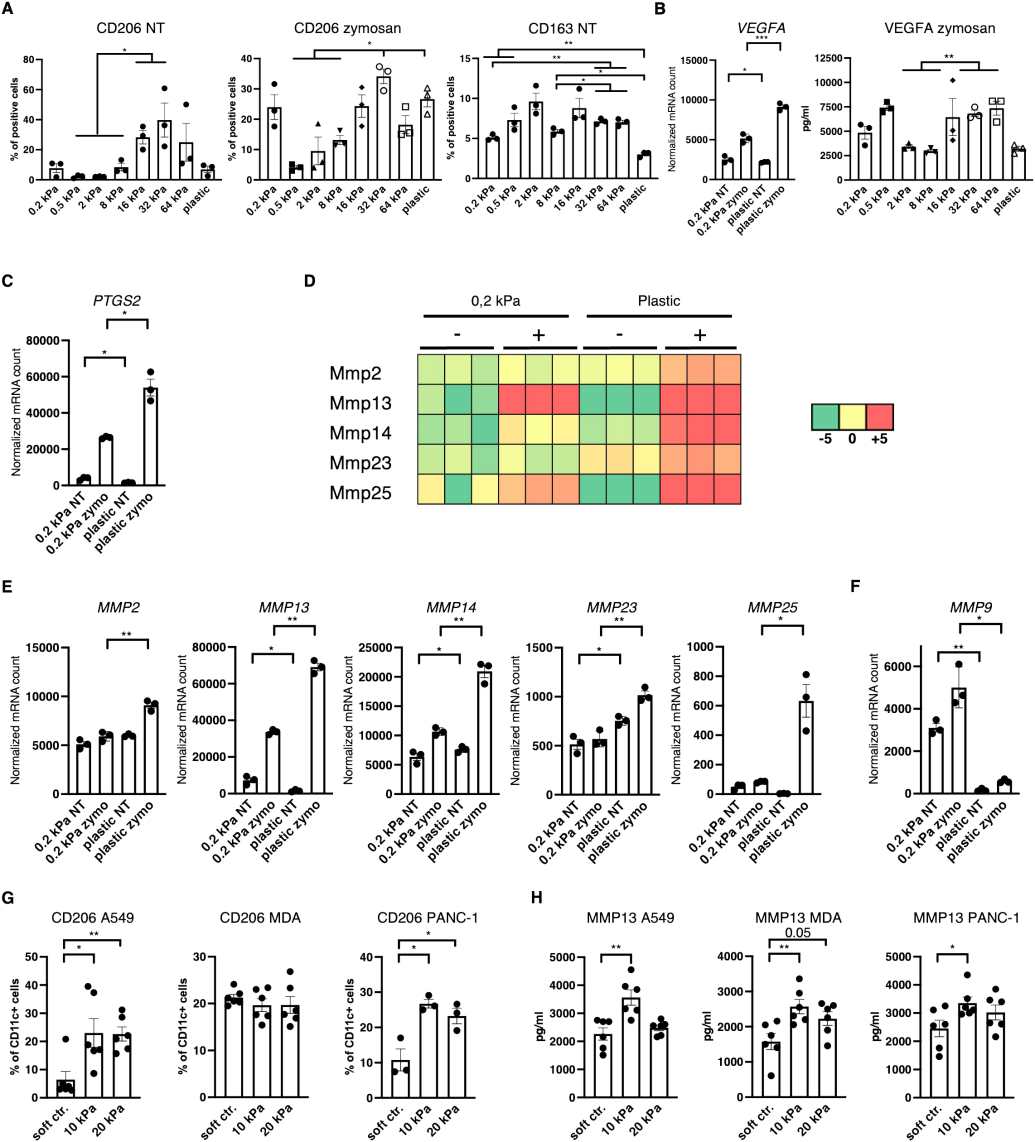
Increased stiffness promotes tumor–associated macrophage marker expression. **(A–F)** Data are derived from murine 2D monocultures. **(A)** CD206^+^ and CD163^+^ cell populations of resting cells (NT, not treated) and zymosan–stimulated cells (24 h) were measured via flow cytometry. **(B)** Normalized *VEGFA* mRNA counts measured during RNAseq and VEGFA production measured via ELISA from zymosan–stimulated (24 h) cells are shown (zymo=zymosan) **(C)** Normalized *PTGS2* (protein also known as COX2) mRNA counts measured during RNAseq are shown. **(D)** Heatmap depicting expression levels of five of the seven highest expressed MMPs of DCs cultured on 0.2 kPa hydrogels (+/- zymosan) and DCs cultured on plastic (+/- zymosan). **(E, F)** Normalized *MMP* mRNA counts measured during RNAseq. **(G)** CD206 expression of CD11c^+^ cells measured via flow cytometry and **(H)** MMP13 production measured via ELISA of human CD14^+^ cell co–cultures. **(G, H)** 10 kPa and 20 kPa were stiffened by addition of CaCl_2_ and soft controls were not stiffened alginate/collagen hydrogels. All experiments, except RNAseq, were performed with three biological repeats per technical repeat and at least two technical repeats in total. RNAseq was performed with one technical repeat. One representative technical repeat is shown in each graph. Three biological repeats per condition were used for the statistical analysis. All statistical differences are shown: *p < 0.05; **p < 0.01; ***p < 0.005; ****p < 0.0001. All error bars are shown as SEM.

Since most myeloid cells in human 3D cancer co–cultures were neither cDC, pDC nor basic macrophages it was hypothesized that most cells would express TAM markers. One of the best established TAM markers is CD206 (24). Indeed, CD206^+^ cell populations increased with stiffness independent of stimulation in 2D murine monocultures (**Figure 5A**). Notably CD163 expression was also increased on intermediate stiffnesses compared to soft substrates but expression decreased on plastic (**Figure 5A**). Additionally, *VEGF* expression and VEGFA production was increased on higher stiffnesses in 2D (**Figure 5B**), along with *PTGS2* expression (**Figure 5C**). Furthermore, out of the seven MMPs that reached mRNA levels around or above 1000 reads, five MMPs were significantly increased on stiffer substrates (**Figures 5D, E**). Out of the seven highest expressed MMPs, *MMP9* was the only gene which was more highly expressed on soft substrates (**Figure 5F**), while *MMP3* expression levels were not significantly different (**Supplementary Figure 1H**). In human cancer co–cultures, CD11c^+^/CD206^+^ populations increased with stiffness for lung and pancreatic cancer co–cultures and reached at least 20% across all cancer types in higher stiffnesses (**Figure 5G**). Furthermore, human MMP13 production increased in 10 kPa compared to soft controls in co–cultures across all cancer types (**Figure 5H**).

### TGF–β and Akt regulate DC and macrophage phenotypes in a context specific way

**Figure 6:**
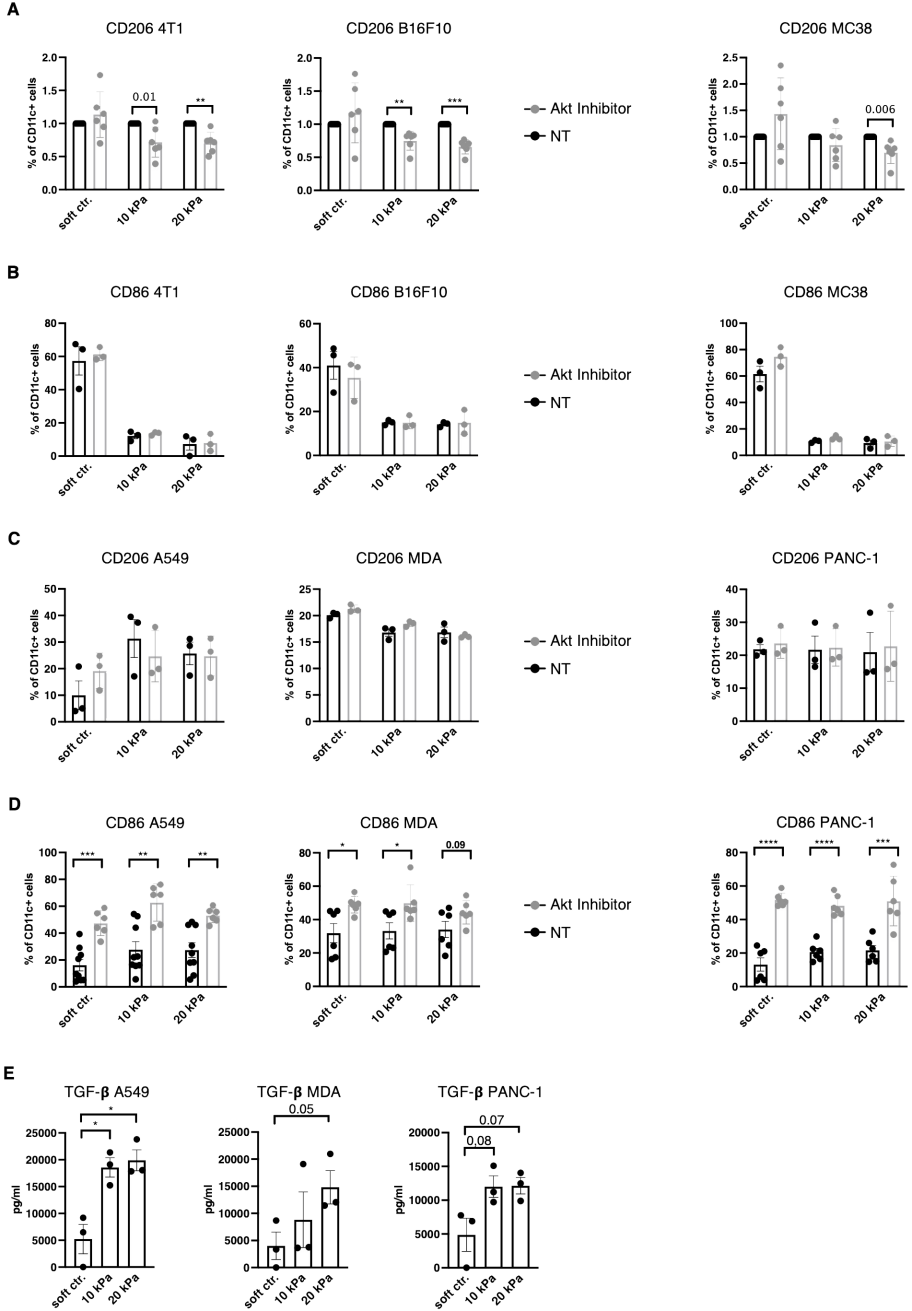
TGF–β and Akt regulates DC and macrophage phenotypes in a context specific way. **(A)** For all graphs, the individual control (NT, not treated) percentages were set to 1 for each stiffness, based on which the corresponding fold change was calculated. **(A)** CD206 and **(B)** CD86 expressions of CD11c^+^ cells were measured via flow cytometry. **(C)** CD206 and **(D)** CD86 expressions of human CD11c^+^ cells were measured via flow cytometry. (**A–D)** Akt was inhibited with 1.2 μM Akt Inhibitor IV (Cayman) for 24 h. **(E)** TGF–β levels were measured via ELISA from supernatant of 3D human cocultures. All experiments were performed with three biological repeats per technical repeat and at least two technical repeats in total. For **(A)** and **(D)** all repeats are shown and were used for the statistical analysis (n = 6 per condition). For **(B, C, E)** one representative technical repeat is shown and three biological repeats per condition were used for the statistical analysis. (**A–E)** 10 kPa and 20 kPa were stiffened by addition of CaCl_2_ and soft controls were not stiffened alginate/collagen hydrogels. All p values are shown: *p < 0.05; **p < 0.01; ***p < 0.005; ****p < 0.0001. All error bars are shown as SEM.

TAM polarization towards M2 has been suggested to be regulated via the PI3K/Akt pathway (39–41) and Akt phosphorylation is reportedly negatively regulated by β2–integrins in DCs (27). Akt was thus hypothesized to be a potential regulator of the DC phenotype to either increase expression of pDC and cDC surface markers in higher stiffness or reduce TAM markers in stiffer substrates. To target the Akt pathway Akt Inhibitor IV (Calbiochem) was used, which targets a kinase upstream of Akt and downstream of PI3K. In murine 3D cancer co–cultures, Akt inhibition indeed led to a decrease in CD11c^+^/CD206^+^ cell populations across all cancer types in stiffer gels (**Figure 6A**). Akt inhibition did not impact the CD11c^+^/CD86^+^ population percentages in murine cultures (**Figure 6B**). In human 3D cancer co–cultures, however, Akt inhibition did not impact CD11c^+^/CD206^+^ populations (**Figure 6C**), while a notable reduction to MMP13 production was observed only in pancreatic cancer co–cultures (**Supplementary Figure 1I**). Instead, Akt inhibition led to a significant increase in CD11c^+^/CD86^+^ populations across all stiffnesses and cancer types (**Figure 6D**). This indicates that Akt is an important regulator of the myeloid phenotype, but its specific regulation mechanism is cell context dependent.

To elucidate the mechanism in human co–cultures independent of intracellular signaling, transforming growth factor beta (TGF–β) levels were measured from culture supernatants. TGF–β has been previously suggested to induce the M2 phenotype (42, 43). Across all human cancer models TGF–β levels increased with stiffness (not all reached significant p values, **Figure 6E**). Thus, DC and macrophages phenotypes might be extracellularly regulated by TGF–β and intracellularly by Akt and this regulation is influenced by stiffness.

## Discussion

The aim of this study was to map the impact of tissue stiffness on DC subset development. Previous reports indicated that integrins, especially β2–integrins, restrict the mature cDC phenotype (27), while a mature cDC phenotype can be induced by blocking overall adhesion of differentiated WT BMDCs or plating them on soft hydrogels overnight (27, 28). However, such phenotypical changes are dynamic and a cDC phenotype that was induced by blocking adhesion begins to reverse six hours after adhesion is re–established (28). What has not been investigated thus far is the outcome of the long–term differentiation on different substrate stiffnesses. The results in this paper show that such cultures result in distinct cDC phenotypes on soft matrices and pDC phenotypes on stiff substrates, as well as functional differences in T cell proliferation, polarization and activation as well as phagocytosis. While the pDC markers CD317, Siglec–H, and TLR7 were all upregulated, B220 was barely expressed. Furthermore, interferon RNA was not detected in cells cultured on stiff substrates. This indicates that high stiffness merely contributes to the pDC phenotype, while other chemical or mechanical signals are required to achieve a functional pDC phenotype.

Nonetheless, this has implications for chronic diseases in which stiffness increases. In this report, this mechanism was investigated in the context of cancer. This study’s results indicate that, in some cancers like melanoma and lung cancer, these pDC–like cells might arise due to stiffness cues, while in other cancers like colon cancer other mechanisms might suppress the expression of pDC markers altogether. What is common across the cancer types investigated, was an increase in macrophage markers, especially TAM markers in humans. While TAMs are well established as promoting tumor progression (24), a CD14^+^ DC subtype has also been previously linked to an immunosuppressive tumor microenvironment (38). Therefore, stiffness could be a hidden contributor to DC dysregulation in cancer.

In this study 3D co–cultures were used, specifically collagen/alginate hydrogels, which were originally established by Elysse C. Filipe et al. (25). To current knowledge, this culture system is the only 3D culture system that allows A) long–term (7+ days) culture without toxicity, B) cell adhesion, C) cell retrieval after culture and D) the generation of matrices of up to 20 kPa without changing the chemical ratio. In terms of immunological studies, one of the biggest drawbacks of this system is the component alginate. Alginate has been previously shown to induce LPS–like immune responses in macrophages (29). Because alginate is a polysaccharide, it is highly likely recognized by CLRs and, for this reason, zymosan was not used for further stimulation on 3D cultures as these cultures were already believed to be stimulated. However similar phenotypes also arose on inert dextran–based hydrogels. Additionally, the use of alginate renders the use of alginate hydrogels *in vivo* inherently flawed.

Nevertheless, this culture system was utilized to identify a potential mechanism via which myeloid cells, specifically DCs, become dysregulated and twisted to support tumor progression by secreting VEGFA and MMPs. Initial attempts to target this mechanism intracellularly were partially successful but revealed an even higher level of complexity. This study confirms other reports of the PI3K/Akt signaling pathway regulating tumor–associated macrophage markers such as CD206 in murine macrophages (40). However, this regulation does not seem to work in human cells in the same way. In human DCs, Akt signaling has been shown to be downstream of CD80/CD86 signaling (44). To current knowledge, the results presented in this study are the first to demonstrate that Akt inhibition led to an increase in CD86 expression in human CD11c^+^ cells. Combined these two studies’ results could indicate that CD86 expression is regulated in a feedback loop in human DCs.

The discrepancy between human and murine Akt inhibition results may be due to a variety of things. One possibility is that variety between human donors was too high and sample size of nine was overall too little to reveal an Akt–dependent regulation of CD206^+^. Another possibility is that intracellular signaling is highly cell context dependent, as the source of DC progenitors differed between the experiments (bone marrow versus blood) and the resulting cell populations might thus have been drastically different. Yet another cell context dependent factor is possible species–specific differences in intracellular signaling. Further studies are needed to explore each possibility.

Nevertheless, higher M2 marker coincided with increased TGF–β secretion in higher stiffness coculture conditions. M2 has been previously suggested to cause M2 polarization (42, 43) and further research is needed to identify potential TGF–β dependent pathways regulating the DC and macrophage phenotypes.

Ultimately, this study found that stiffness governs DC and macrophage phenotypes, a mechanism which possibly contributes to DC and macrophage dysregulation in cancer. How exactly these phenotypes are regulated intracellularly remains unclear, but the mechanism seems to be highly cell context dependent. Similar differences might exist in general cell signaling and contribute to most drugs identified on the bench failing during clinical trials (45). Nevertheless and in any context, Akt was identified as an important regulator of DC and macrophage surface marker expression. This is relevant, because both reduction of TAM markers and increase of CD86 expression might be beneficial for increased patient survival and targeting Akt could be a viable strategy to achieve this.

## Data Availability

The datasets presented in this study can be found in online repositories. The names of the repository/repositories and accession number(s) can be found below: Sequence Read Archive under the Accession number: PRJNA1082503.
