# Supplementary material for: Stiffness regulates dendritic cell and macrophage subtype development and increased stiffness induces a tumor–associated macrophage phenotype in cancer co–cultures
Source: Front Immunol. 2024 Aug 15;15:1434030. doi: 10.3389/fimmu.2024.1434030 (PMC11358102; doi:10.3389/fimmu.2024.1434030)
Supplement: Supplementary file 1 [file DataSheet1.pdf]

Supplementary Figure

Supplementary Figure 1

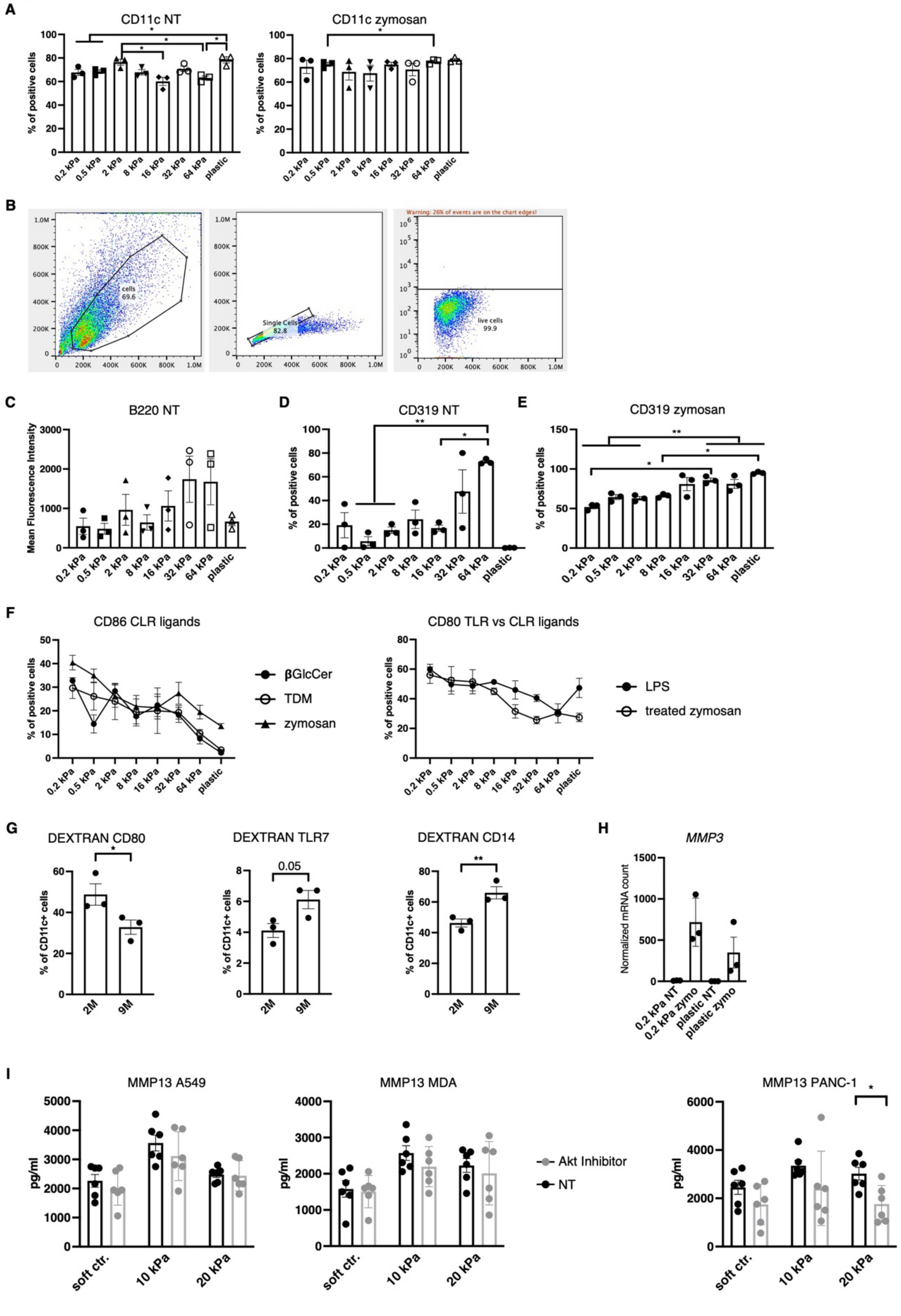

Supplementary Figure 1

A) CD11c$^+$ cell populations were measured via flow cytometry on resting cells (NT, not treated) and zymosan-stimulated cells. Percentages of all living cells are shown. **B)** Gating strategy for flow cytometry measurements. **C)** The mean fluorescence intensity (MFI) of B220 from resting BMDC cultures (NT, not treated) measured via flow cytometry. **D)** Percentages of resting and **E)** zymosan stimulated BMDCs positive for CD319 were measured via flow cytometry. **F)** CD86 expressions as an example of surface marker expression after stimulation with Mincle DAMP ligand (β-glucosylceramide [beta GlcCer]) and PAMP (trehalose 6,6′ -dimycolate [TDM]) along with Dectin-1 and TLR4 ligand zymosan (left). On the right, stimulation with LPS (TLRs) compared to treated zymosan (Dectin-1 only) is depicted, for which CD80 expression was measured. **G)** The cell populations of BM-derived cells cultured in 3D dextran hydrogels. Surface marker expression was measured via flow cytometry. Cell populations were first gated for CD11c in order to exclude gel particles and the graphs depict the percentage of CD11c$^+$ cells. 2M means 2mM and the stiffness is roughly 2kPa, while 9M means 9mM and the stiffness should roughly be 9kPa. **H)** Normalized MMP3 mRNA counts measured during RNAseq are shown (NT, not treated; zymo = zymosan). RNAseq was performed with murine BMDCs cultured on 2D hydrogels and compared to plastic. **I)** MMP13 production was measured via ELISA of human CD14$^+$ cell co-cultures. The statistical differences are shown as $*p < 0.05$; 10 kPa and 20 kPa were stiffened by the addition of $CaCl_2$ and soft controls were not stiffened alginate/collagen hydrogels. Akt was inhibited with 1.2 μM Akt Inhibitor IV (Cayman) for 24 h. **A–I)** Experiments were performed with three biological repeats per technical repeat and at least two technical repeats in total (except RNAseq).
